# RETRACTION: Impact of Diabetic Versus Non‐Diabetic Patients Undergoing Coronary Artery Bypass Graft Surgery on Postoperative Wound Complications: A Meta‐Analysis

**DOI:** 10.1111/iwj.70363

**Published:** 2025-03-17

## Abstract

**RETRACTION:** HeL. , LiuM. , HeY. , and GuoA. , “Impact of Diabetic Versus Non‐Diabetic Patients Undergoing Coronary Artery Bypass Graft Surgery on Postoperative Wound Complications: A Meta‐Analysis,” International Wound Journal 21, no. 3 (2024): e14495, 10.1111/iwj.14495.37989726 PMC10898396

The above article, published online on 21 November 2023, in Wiley Online Library (http://onlinelibrary.wiley.com/), has been retracted by agreement between the journal Editor in Chief, Professor Keith Harding; and John Wiley & Sons Ltd. Following an investigation by the publisher, all parties have concluded that this article was accepted solely on the basis of a compromised peer review process. The editors have therefore decided to retract the article. The authors did not respond to our notice regarding the retraction.

**RETRACTION:** L. He , M. Liu , Y. He , and A. Guo , “Impact of Diabetic Versus Non‐Diabetic Patients Undergoing Coronary Artery Bypass Graft Surgery on Postoperative Wound Complications: A Meta‐Analysis,” International Wound Journal 21, no. 3 (2024): e14495, 10.1111/iwj.14495.37989726 PMC10898396

The above article, published online on 21 November 2023, in Wiley Online Library (http://onlinelibrary.wiley.com/), has been retracted by agreement between the journal Editor in Chief, Professor Keith Harding; and John Wiley & Sons Ltd. Following an investigation by the publisher, all parties have concluded that this article was accepted solely on the basis of a compromised peer review process. The editors have therefore decided to retract the article. The authors did not respond to our notice regarding the retraction.

